# Oxidative Stress in the Rostral Ventrolateral Medulla Contributes To Cardiovascular Regulation in Preeclampsia

**DOI:** 10.3389/fphys.2017.00772

**Published:** 2017-10-04

**Authors:** Jiu-Qiong Yan, Fang Huang, Fan Hao, Xiao-Ling Su, Qi Meng, Ming-Juan Xu

**Affiliations:** ^1^Department of Obstetrics and Gynecology, Changhai Hospital, Second Military Medical University, Shanghai, China; ^2^Department of Geriatics, Jinling Hospital, Nanjing, China

**Keywords:** preeclampsia, rostral ventrolateral medulla, reactive oxygen species, sympathetic overactivity, blood pressure

## Abstract

**Background:** It has been demonstrated that preeclampsia, a pregnancy-specific hypertension disorder, is characterized by high blood pressure (BP) and sympathetic overactivity. Increased reactive oxygen species (ROS) in the rostral ventrolateral medulla (RVLM), a key region for controlling sympathetic tone, has been reported to contribute to high level of BP and sympathetic outflow. The aim of the present study was to determine the role of the RVLM ROS in mediating the preeclampsia-associated cardiovascular dysfunction.

**Methods:** The animal model of preeclampsia was produced by administration of desoxycorticosterone acetate (DOCA) to pregnant rats.

**Results:** Compared with normal pregnant rats without DOCA treatment (NP), the protein concentration and norepinephrine excretion in 24-h urine, as well as BP in pregnant rats with DOCA treatment (PDS) were significantly increased. The levels of superoxide anion and the protein expression of NADPH oxidase subtype (NOX4) in the RVLM were significantly increased in PDS than in NP groups. Furthermore, microinjection of the superoxide dismutase (SOD) mimic Tempol (5 nmol) into the RVLM significantly decreased BP, heart rate, and renal sympathetic never activity in PDS but not in NP group.

**Conclusion:** The present data suggest that high BP and sympathetic overactivity in preeclampsia rats is associated with increased oxidative stress in the RVLM via upregulation of NOX4 expression.

## Introduction

Preeclampsia is a pregnancy-specific disorder with the *de novo* onset of hypertension and proteinuria after 20 weeks of gestation (Pridjian and Puschett, 2002a,b). This disease has become to be a major cause resulting in morbidity and mortality in pregnant women and neonates (Lenfant, 2001; Roberts et al., 2016). In additional to high blood pressure (BP), preeclampsia has been reported to exhibit an obvious sympathetic overactivity (Fischer et al., 2004; Jarvis et al., 2012; Logue et al., 2016). Furthermore, a prospective study in pregnant women has revealed that pregnancy-associated with sympathetic overactivity precedes preeclampsia (Fischer et al., 2004). However, the underlying mechanism of increased sympathetic outflow involved in cardiovascular dysfunction in preeclampsia remains unclear.

Sympathetic nerve overactivity is recognized as an important mechanism responsible for pathological process of cardiovascular diseases such as hypertension and chronic heart failure (Greenwood et al., 2003; Dell'Oro et al., 2017). It is well-known that the rostral ventrolateral medulla (RVLM) plays a key role in mediating tonic and reflex control of the cardiovascular activity (Dampey, 1994; Guyenet, 2006). RVLM has been widely accepted to be involved in maintaining resting BP and sympathetic tone (Guyenet, 2006). Moreover, accumulating studies indicate that oxidative stress, which is produced by the imbalance between mechanisms of reactive oxygen species (ROS) production (e.g., NADPH oxidase) and mechanisms of ROS clearance (e.g., superoxide dismutase, SOD), contributes to the enhanced central sympathetic outflow in the RVLM in experimental animal models of hypertension and chronic heart failure (Peterson et al., 2006; Hirooka et al., 2011). Interestingly, oxidative stress in peripheral system has been reported to be associated with the pathophysiology of preeclampsia (Siddiqui et al., 2010; Guerby et al., 2015). For example, the oxidative stress level in serum of patients was increased during preeclampsia (Medrano Rodriguez et al., 2008; Genc et al., 2011), while an reduction in the oxidative stress in placentas significantly improves N-omega-nitro-L-arginine methyl ester (L-NAME)-induced preeclamptic symptoms in the preeclamptic Sprague-Dawley (SD) rat model (Xuan et al., 2016). However, there is no direct evidence showing the relationship between oxidative stress in the RVLM and cardiovascular dysfunction including high BP and sympathetic overactivity in preeclampsia.

Therefore, the main goal of this study was to determine the role of the oxidative stress-mediated sympathetic overactivity in cardiovascular dysfunction in preeclampsia. First, we determined the level of oxidative stress in the RVLM in rat model with preeclampsia. Second, we detected the possible sources of oxidative stress in the RVLM in preeclampsia. Finally, we observed the effects of microinjection of Tempol (superoxide dismutase mimic) into the RVLM on cardiovascular activity in preeclampsia.

## Methods

### Experimental protocols

A total of 90 rats were used in this study. Female SD rats (200–250 g) and male SD rats (275–300 g) were supplied by Sino-British SIPPR/BK Laboratory Animal Ltd (Shanghai, China). All experimental procedures were approved by the Institutional Care and Use Committee of the Second Military Medical University.

### Animal model with preeclampsia

Desoxycorticosterone acetate plus increased NaCl intake produced hypertension, proteinuria, rapid weight gain, convulsions, decreased litter size, decreased offspring weight, increased fetal and maternal mortality, and renal lesions similar to those seen in human preeclampsia (Douglas, 1976). Therefore, the animal model of preeclampsia was produced by administration of DOCA to pregnant rats. The female animals were mated with male SD rats. Pregnancy of female rats was confirmed by vaginal smearing. The animals were divided into four groups: Group1 was non-pregnant animals (control); Group2 was non-pregnant animals treated with DOCA and saline (NPS). On based on previous study (Ianosi-Irimie et al., 2005; Uddin et al., 2010; Moraloglu et al., 2012), NPS group was injected initially with 12.5 mg of DOCA intraperitoneally in a depot form, followed by 6.5 mg on a weekly basis. Their drinking water was replaced with 0.9% saline. Group3 was normal pregnant without DOCA treatment (NP). Pregnant animals were given by tap water and libitum. Group 4 was normal pregnant treated with DOCA and saline (PDS). All 4 groups were maintained on normal rat chow. At 18–19 days of pregnancy, 24 h-urine was collected in the absence of food. Each animal was housed separately in a metabolic cage. Before the animals euthanized by pentobarbital sodium (200 mg/kg, i.p.), BP and heart rate (HR) were measured. At the end of experiment, the brain was quickly removed for further analysis.

### General surgery, recording of RSNA, and RVLM microinjection

The protocol for RVLM microinjection was based on previous study (Peng et al., 2011). Briefly, rats were anesthetized by intraperitoneal injection of urethane (800 mg/kg) and a-chloralose (40 mg/kg). The trachea of rats was cannulated for mechanical ventilation. The right femoral artery was catheterized for measuring BP and HR via a Powerlab system. The procedure for recording of RSNA was described previously (Peng et al., 2009). The left renal sympathetic nerves were exposed, identified and dissected free of the surrounding connective tissue, and placed on a pair of recording electrodes. Both the nerve and the electrodes were covered with a fast-setting silicone (Wacker Sil-Gel). The signal was amplified, monitored, and analyzed by Powerlab system. Noise levels were subtracted from the nerve recording data before percent changes from baseline were calculated. Integrated RSNA was normalized as 100% baseline in the control period.

The anesthetized rats were placed in a stereotaxic frame, and the dorsal surface of the medulla was surgically exposed. Microinjections were made from a three-barrel micropipette (20–50 μm diameter). According to rat atlas (Paxinos and Watson, 1998), the coordinates for RVLM were 2.0–2.5 mm rostral to obex, 1.8–2.1 mm lateral to midline, and 2.8–3.2 mm ventral to the dorsal surface of the medulla. The injection was made over a period of 5–10 s and the injection volume (100 nl) was carefully measured by observing the movement of the fluid meniscus along a reticule in a microscope. The RVLM was chemically identified by a pressor response (>20 mmHg) to L-glutamate (1 nmol) microinjection. The changes in BP, HR, and renal sympathetic nerve activity (RSNA) were recorded 10 min after injection of Tempol (5 nmol), which was based on the previous study (Wang et al., 2016; Zahid et al., 2016). At the end of each experiment, 100 nl of 2% pontamine sky blue solution was injected into the RVLM to mark the site. The injection sites in the study were confirmed to be located within the RVLM.

### Western blot analysis

Rats were euthanized and the brains were removed for western blot analysis. The protocol for Western blot analysis was described in our previous study (Hao et al., 2016). In brief, the rat brains were removed and immediately frozen, blocked in the coronal plane, and sectioned at 100 μm thickness in a cryostat from 2.0 to 3.0 mm rostral to the Obex. The total span of punches was 1 mm, from 1.6 to 2.6 mm lateral to the midline. The RVLM was then punched using a punch-needle (0.6 mm inside diameter) from 10 sections. The punched tissues from the 10 sections were pooled for protein extraction. The protein concentration was measured with BCA kit. The protein samples were loaded onto a 10% SDS–PAGE gel and then transferred to PVDF membrane. One lane on the right contained a molecular weight marker, which provides a visual check of transfer efficiency and marks band position for target protein. The membrane was incubated with primary antibody (SOD1 rabbit anti-mouse, Santa Cruz, sc-650, 1:2,000; NOX4 rabbit anti-mouse, Santa Cruz, sc-650, 1:1,000) and secondary antibodies (1:10,000). The protein bands were visually detected and analyzed. The levels of target proteins were normalized to tubulin, which served as a loading control.

### Measurement of urinary norepinephrine excretion by high-performance liquid chromatography (HPLC)

Urinary norepinephrine content was measured using HPLC at the 18–19 days of pregnancy. HPLC (model 582 pump, ESA) with electrochemical detection (model 5300, ESA) was used to detect content of norepinephrine (NE) in 24 h urinary excretion, as described previously (Peng et al., 2011). Briefly, 24-h urinary samples were collected after rats were placed in metabolism cages for 24 h and acidified with glacial acetic acid in 15-ml centrifuge tubes, which were embedded in crushed ice. Dihydroxybenzylamine (Sigma) was used as the internal standard. NE was absorbed onto acid-washed alumina with 3 mol/L Tris (hydroxymethyl) aminomethane buffer at pH 8.6 in 2% EDTA. The alumina was then washed three times with 3 ml of distilled water. NE was extracted with 400 μl of 0.2 M glacial acetic acid with 5 min of shaking and a final 30-min settlement. The supernatant (50 μl) was injected into the HPLC column [reverse phase, ESA, 150 × 3.2 mm, 3 um C18 (P/N70–0636)], and NE was eluted with the mobile phase (80 mM citric acid monohydrate, 73.4 mM citric acid trisodium salt, 0.12 mM 1-octanesulfonic acid sodium salt, and 0.1 mM EDTA adjust to pH 4.3 with phosphoric acid). The flow rate was set at 0.5 ml/min.

### Measurement of ROS generation in the RVLM

We performed fluorescence microtopography to detect ROS content in the RVLM as previously described (Peng et al., 2009). The rats were euthanized and perfused through the aorta with 0.9% NaCl solution. The brain stem were removed and rapidly frozen. According to the rat atlas (Paxinos and Watson, 1998), the target area RVLM sections of 10 μm thickness were cut in a cryostat, and placed on glass slides and incubated at room temperature in the dark for 30 min with Dihydroethidium (DHE, 5 μmol/L). DHE, an oxidant-sensitive probe, is widely used for detection of ROS. Two products of DHE oxidation, ethidium, and 2-hydroxyethidium, can bind to the nuclear DNA, thereby forming a strong red fluorescent complex. The reaction usually takes place about 30 min (He et al., 2011; Zhou et al., 2013). The microslide with brain slice were washed three times 5 min in PBS (0.01 mol/L, pH 7.4). Fluorescence was determined using a microscope (Nikon, Japan) at excitation and emission wavelengths of 535 and 590 nm, respectively. Fluorescence intensity was semi-quantitatively analyzed by Leica LAS-AF lite 2.1.1. A total of five rats were used to measure ROS in the RVLM by DHE each group. Fluorescence value of each animal was detected by a brain slice at the same Bragma parameter (–12.24 mm) for statistical analysis.

Furthermore, brain tissues the RVLM was punched on coronal sections and was further performed to detect the ROS level in the RVLM by the GENMED lucigenin chemiluminescence quantitative determination kit (GEMED, GSM101113.5, USA). These procedures were performed according to the manufacturer's instruction. In brief, after RVLM tissue was punched and weighed from the rat which was euthanized (pentobarbital sodium, 300 mg/kg, i.p.), 80 μL Protein Lysis Buffer (Cell Signaling Technology, USA) was added into the test tube, tissue was polished by electric homogenizer and then centrifuging for 20 min. Supernatant was collected for analysis by lucigenin chemiluminescence quantitative assay. Chemiluminescence was expressed as arbitrary units.

### Measurement of urinary protein concentration in 24 h urine

Urine was collected for 24 h urinary protein quantization. The 24-h protein excretion was measured using the enzyme-linked immunosorbent assay kit (purchased from Beyotime Biotechnology, China). Measurement of urinary protein concentration was completed according to the manufacturer's instructions. In brief, 24 h urine was collected for each rat, Protein concentration was determined using Bradford method with Bradford Protein Assay. Absorbance was measured at 595 nm with Microplate Reader and BSA was used as standard.

### Statistical analysis

All values in this study were expressed as mean ± SEM. Statistical comparisons between different groups were made by the one-way ANOVA, and followed by Bonferroni's *post-hoc* analysis. All analyses were performed by software (SPSS18.0). A value of *P* < 0.05 was considered statistically significant.

## Results

### Cardiovascular dysfunction in preeclampsia rats

**Figure 1:**
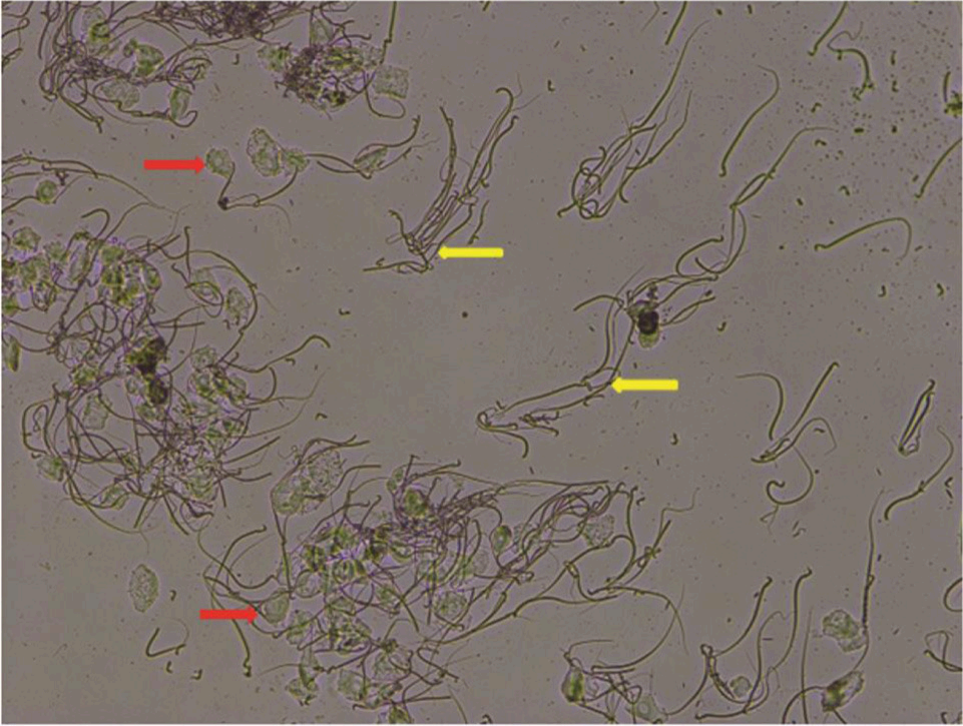
Pregnancy was confirmed by vaginal smearing. Vaginal epithelial cells (red arrow) and male sperm (yellow arrow) were observed in vaginal smear after rats mating, magnification: × 100.

**Figure 2:**
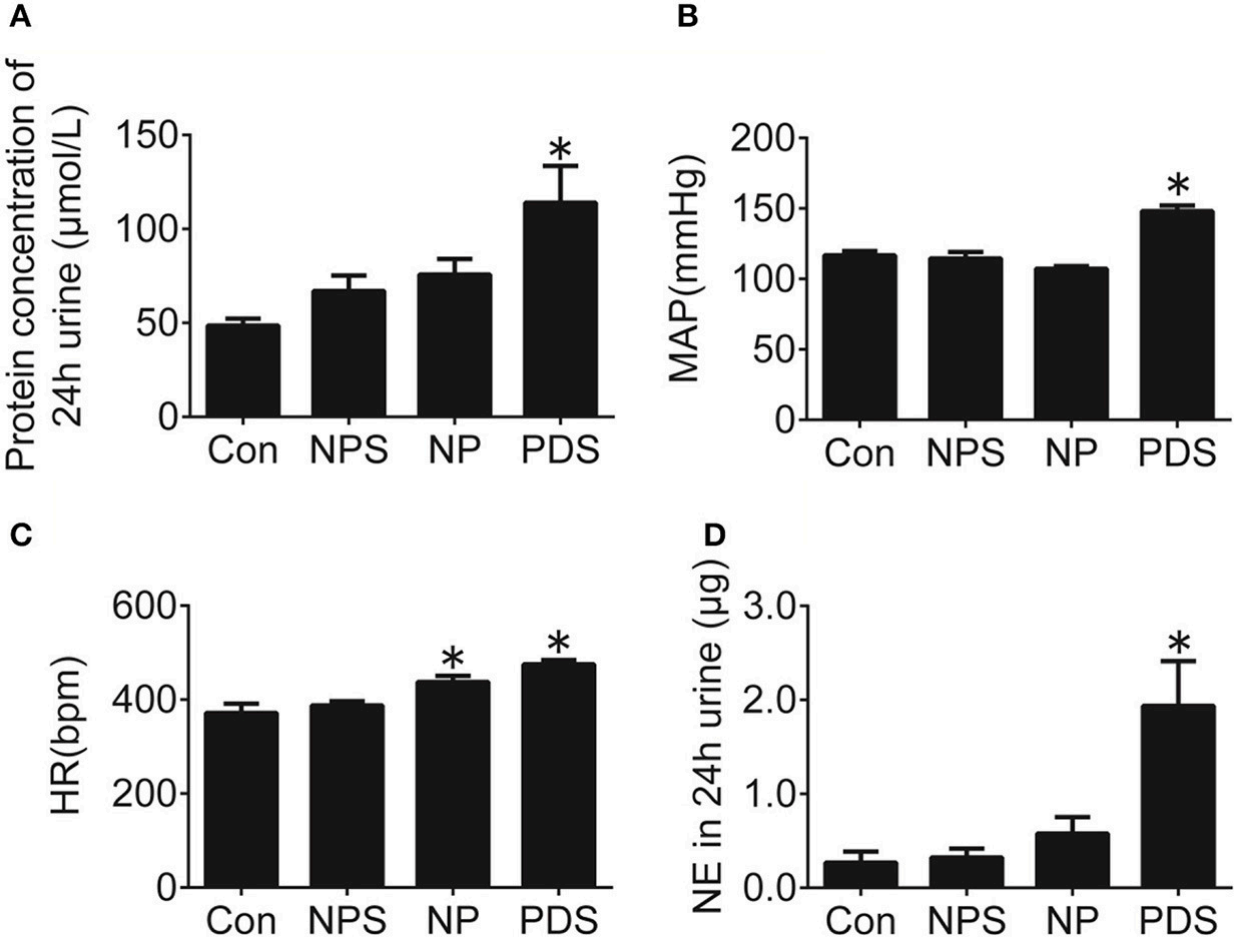
Cardiovascular dysfunction in preeclampsia rats. Protein concentration in 24-h urine **(A)**, MAP **(B)**, HR **(C)**, and NE in 24-h urine **(D)** of four groups. *n* = 5/group, ^*^p < 0.05 vs. Con group.

Rats with pregnancy were confirmed by vaginal smearing at first day after mating (Figure 1). At 18 days of pregnancy, PDS group showed a significant increase in concentration of urinary protein (114 ± 19.6 vs. 48.5 ± 3.76 μmol/L, *p* < 0.05), mean arterial pressure (MAP, 148 ± 3.99 vs. 117 ± 3.03 mmHg, *p* < 0.05), HR (475 ± 9.01 vs. 371 ± 19.3 bpm, *p* < 0.05), and the content of norepinephrine in the 24 h urine (1.94 ± 0.477 vs. 0.269 ± 0.117 μg, *p* < 0.05; Figure 2) compared with the rats in Con group. However, these parameters were no significant differences between NPS group and NP group.

### The level of oxidative stress in the RVLM in preeclampsia rats

**Figure 3:**
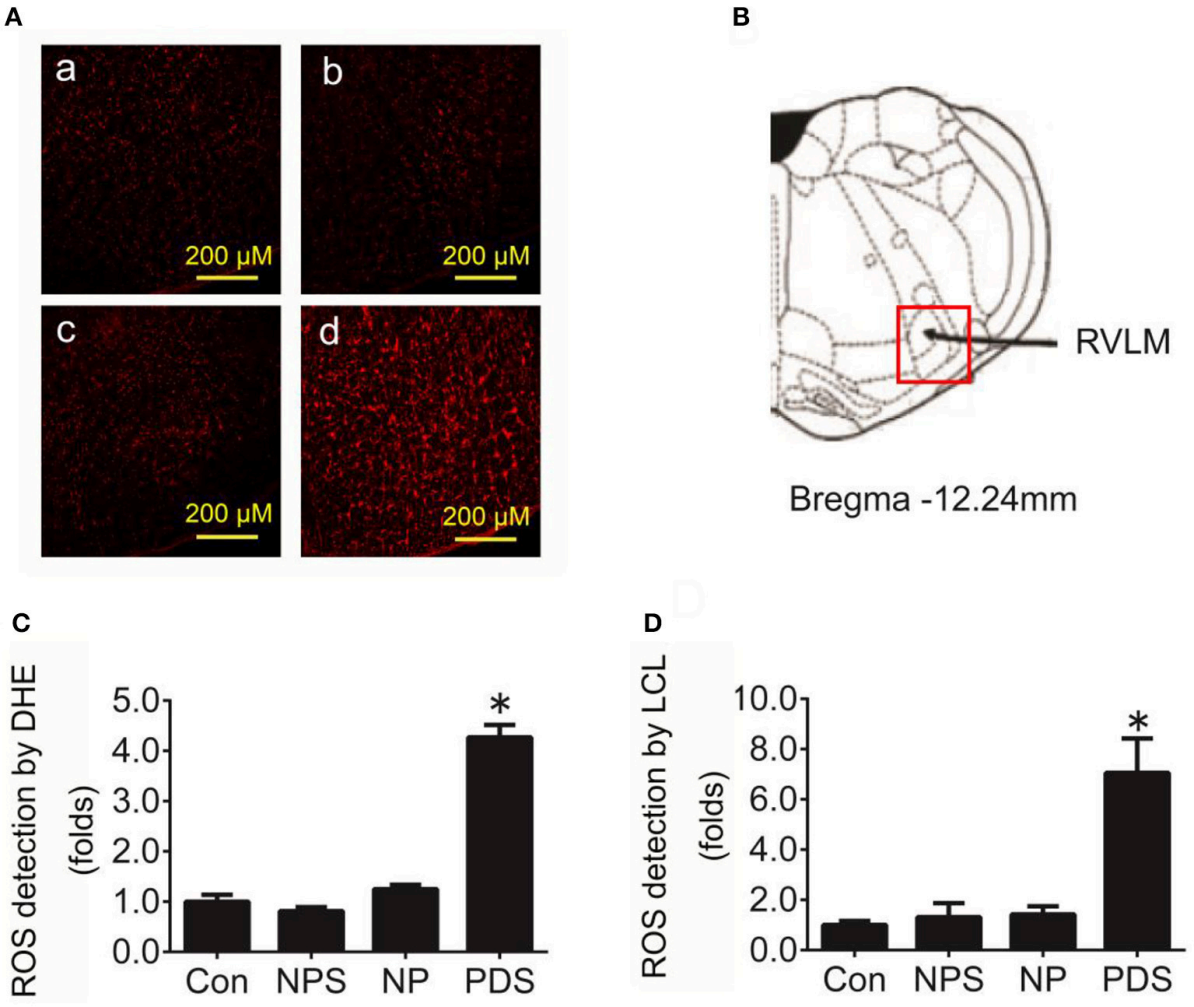
The level of oxidative stress in the RVLM in preeclampsia rats. **(A)** Representative confocal images of ROS in the RVLM stained by DHE fluorescent labeling (red) in four groups, Con group (a), NPS group (b), NP group (c), and PDS group (d). Scale bar, 200 μm. **(B)** Location of RVLM region in the standard alts of rat brain; **(C)** Histogram shows the fluorescence intensity of DHE fluorescence staining of the RVLM in different groups. *n* = 5/group, ^*^p < 0.05 vs. Con group. **(D)** Quantification of superoxide anion content in the RVLM detected by lucigenin chemiluminescence (LCL) in four groups. *n* = 5/group, ^*^p < 0.05 vs. Con group.

**Figure 4:**
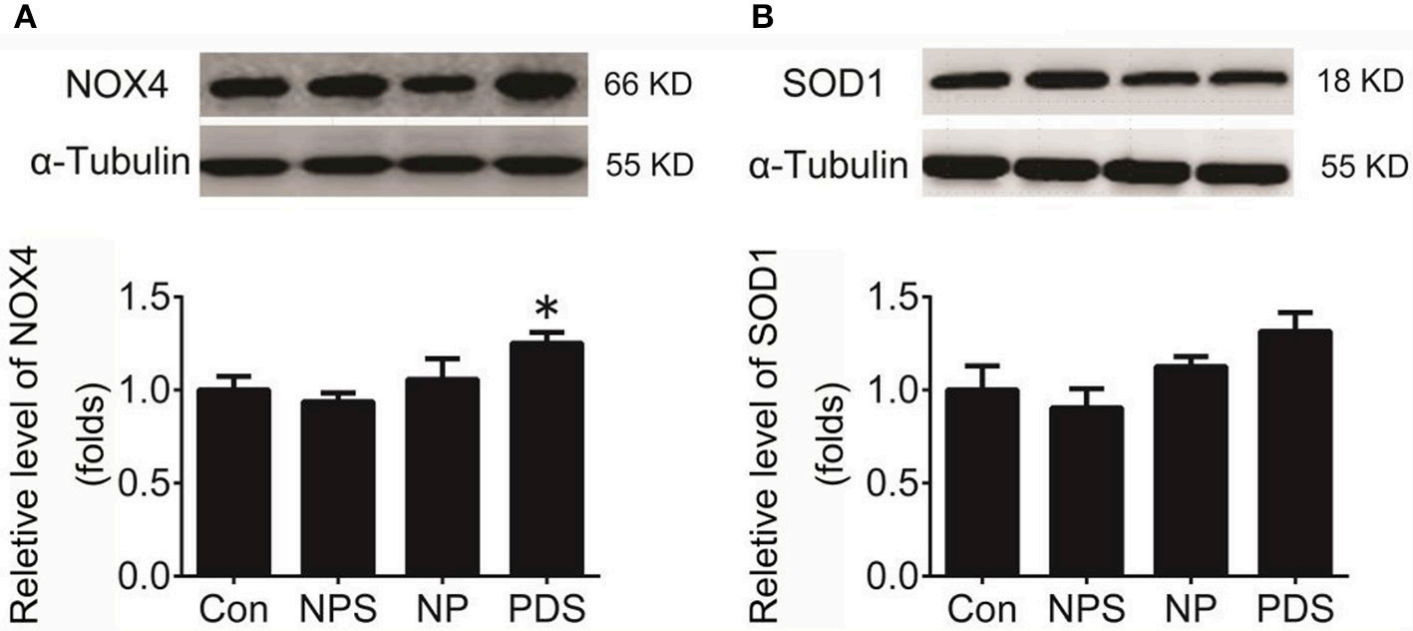
Protein expression of NOX4 and SOD1 in the RVLM. **(A)** The representative original protein bands (top) and quantification (bottom) of target protein NOX4 expression in the RVLM of four groups; *n* = 5/group, ^*^p < 0.05 vs. Con group; **(B)** The representative original protein bands (top) and quantification (bottom) of SOD1 protein expression in the RVLM of four groups; *n* = 5/group, ^*^p < 0.05 vs. Con group.

As indicated in Figure 3, it was shown that the ROS content in the RVLM using DHE probe in PDS group was increased remarkably compared with Con groups (4.27 ± 0.248 vs. 1.00 ± 0.134, *p* < 0.05). Furthermore, we used the tissue super oxide anion lucigenin chemiluminescence quantitative detection kit to detect the ROS level in the RVLM, it was observed that ROS levels in RVLM was significantly (*p* < 0.05) higher (≈7-fold) in PDS group compared with Con groups. As shown in Figure 4, the protein expression of NOX4 in the RVLM was upregulated in PDS group compared with Con group. The protein expression of SOD1 had no significant changes in the RVLM of PDS group compared with Con group. In additional, the protein expression of NOX4 and SOD1 between NPS group and NP group also had no significant differences.

### The effects of microinjection of tempol into the RVLM on cardiovascular activity in preeclampsia

**Figure 5:**
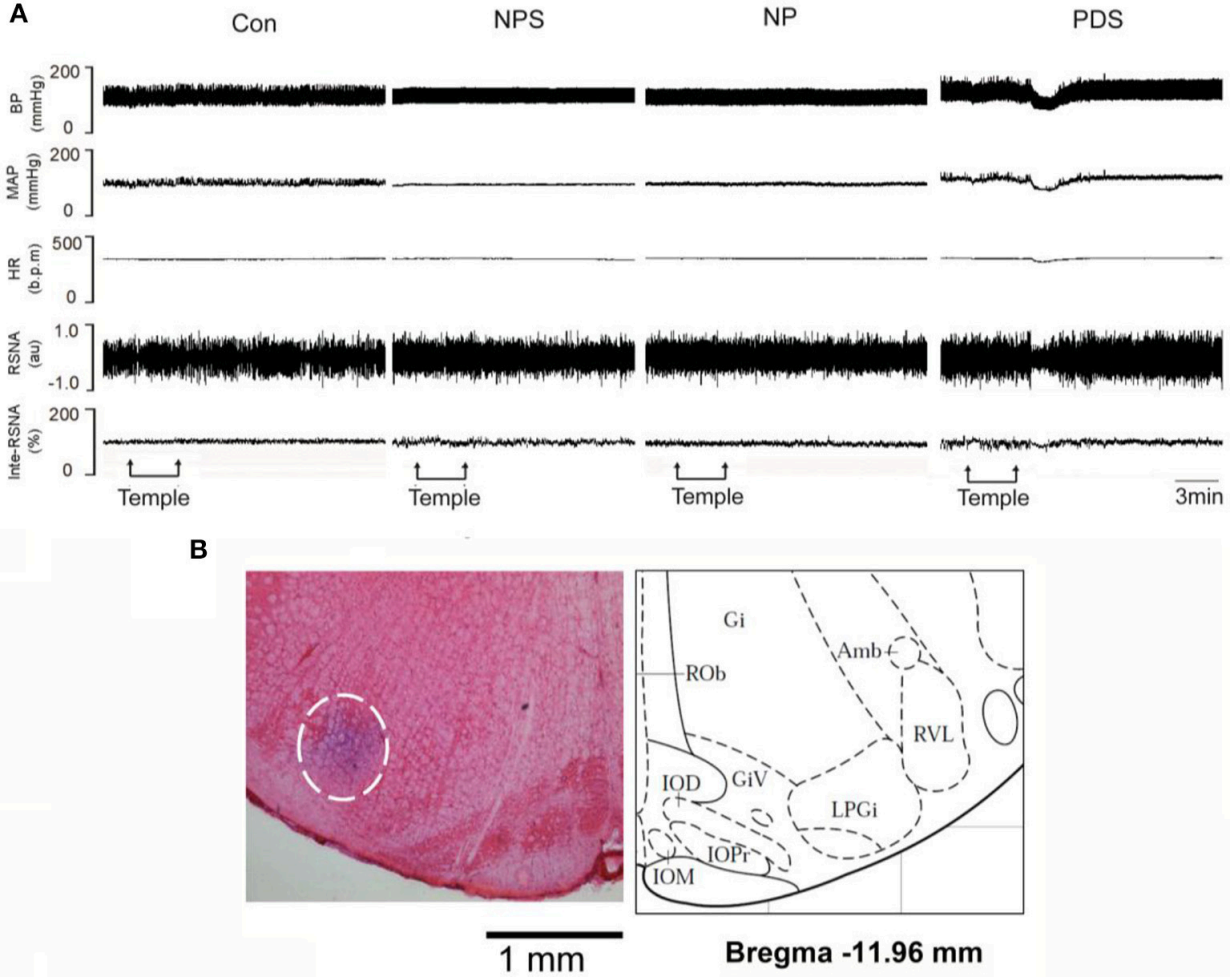
The effects of RVLM microinjection of Tempol on cardiovascular activity in preeclampsia. **(A)** Representative traces showing the responses of MAP, HR, and RSNA to microinjection of Tempol (5 nmol) into the RVLM in four groups. **(B)** The original picture showing the RVLM microinjection site stained by pontamine sky, and the standard alts of RVLM region in rat brainstem.

**Figure 6:**
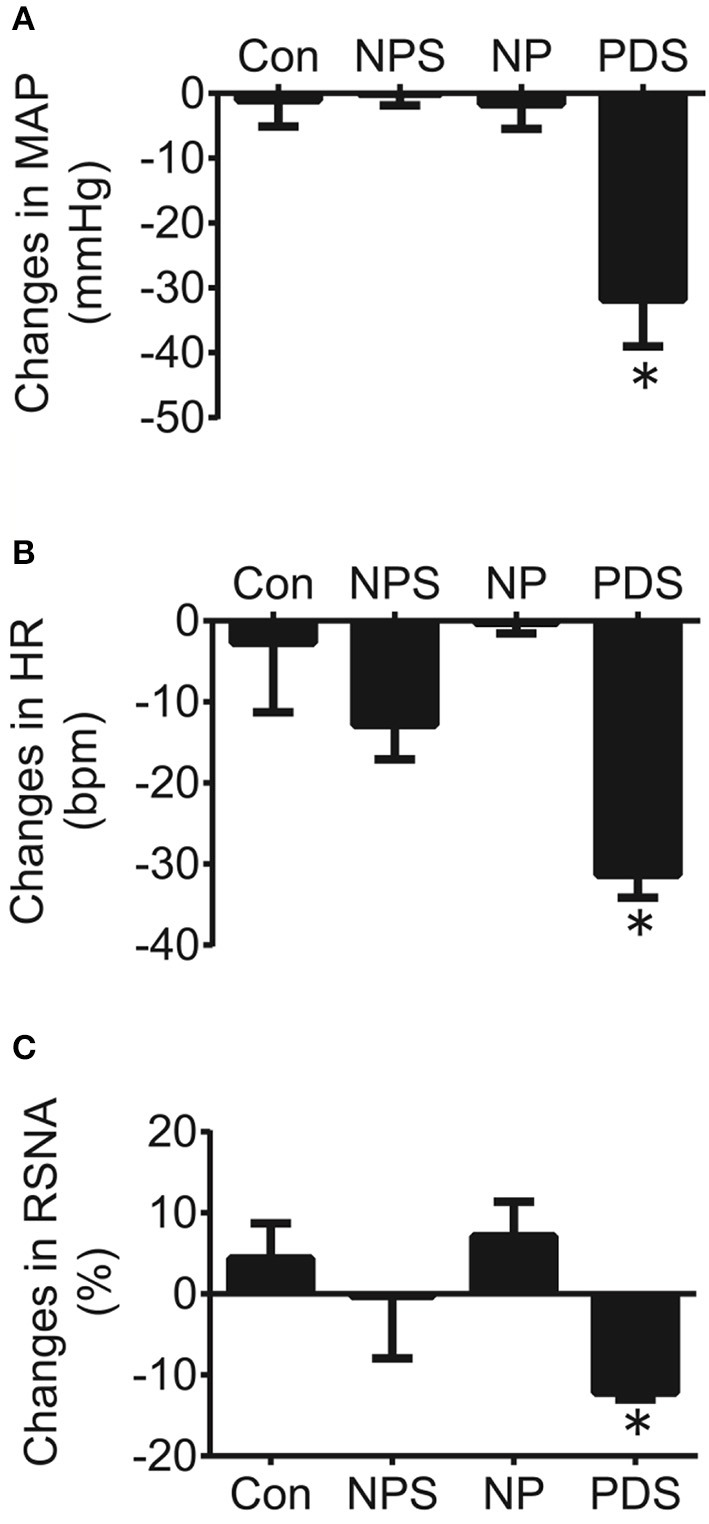
The peak changes in MAP **(A)**, HR **(B)**, and RSNA **(C)** induced by microinjection of Tempol (5 nmol) into the RVLM in four groups. *n* = 5/group. ^*^P < 0.05 vs. Con.

The baseline BP and HR in four groups for microinjection of Tempol into the RVLM was shown in Table 1. As shown in Figures 5, 6, microinjection of Tempol (5 nmol) into the RVLM significantly decreased MAP (–31.8 ± 5.09 vs. –1.06 ± 2.02 mmHg, *p* < 0.05), HR (–31.3 ± 2.00 vs. –2.72 ± 4.29 bpm, *p* < 0.05), and RSNA (–12.2 ± 0.609 vs. 4.38 ± 2.17 %, *p* < 0.05) in PDS group compared with Con group. The peak changes in MAP, HR, and RSNA induced by microinjection of Tempol into the RVLM between NPS group and NP group has no significant difference.

**Table 1:** Baseline values of BP and HR before microinjection of Tempol into the RVLM in four group rats.

**Group**	**BP (mmHg)**	**HR (bpm)**
Con	116 ± 5	375 ± 13
NPS	118 ± 4	372 ± 12
NP	124 ± 6	368 ± 17
PDS	143 ± 3^*^	376 ± 14

*Data are presented as mean ± SEM, bpm, beats per minute. n = 5/group*,

* *p < 0.05 vs. Con*.

## Discussion

The major findings of the present study were: (1) The rat model of preeclampsia by administrating DOCA and saline showed an increase in MAP, HR, and the content of norepinephrine in the 24 h urine; (2) the ROS level in the RVLM in preeclampsia rats was significantly increased; (3) the protein expression of NOX4 in the RVLM was up-regulated in preeclampsia rats; (4) microinjection of Tempol into the RVLM significantly decreased BP, HR, and RSNA in preeclampsia rats. On the basis of these results, it is suggested that increased oxidative stress in the RVLM contributes to high BP and sympathetic overactivity in preeclampsia.

In this study, the rat model of preeclampsia was successfully established by administrating DOCA and saline water to pregnant rats, as described in previous study (Ianosi-Irimie et al., 2005). Although, many animal models share features with the hypertensive disorders of pregnancy, most of them do not include the complete spectrum of symptoms present in the human disease. It has been suggested that the increase of extracellular fluid can lead to hypertension, which is due to the activation of the renin-angiotensin system (RAS; Kobori et al., 2007). All of the RAS components are present and intra-renal angiotensin II (Ang II) is formed by independent multiple mechanism. Ang II exerts a cardinal role in the pathogenesis of hypertension (Kobori et al., 2007). Many studies have reported associations between RAS and preeclampsia (Li et al., 2016; Aung et al., 2017), indicating that RAS may also play a role in the pathogenesis of preeclampsia. Although, the peripheral RAS (plasma levels of active renin, renin and Ang II) is downregulated in the DOCA preeclampsia model (Uddin et al., 2010); the association of gene polymorphisms of RAS and preeclampsia was observed in human, moreover, T allele of angiotensinogen may involve in the pathogenesis of PE (Aung et al., 2017). During pregnancy, an increase in extracellular fluid volume occurs, which reaches a 40–50% increase by the end of gestation (Scott, 1972; Gallery and Brown, 1987). Since pregnancy represents a condition in which spontaneous volume expansion of the extracellular fluid occurs (Scott, 1972; Gallery and Brown, 1987). It has also been reported that the burden of volume expansion represented by pregnancy would be sufficient to cause the development of hypertension (Ianosi-Irimie et al., 2005). Therefore, the rat model of preeclampsia by administrating DOCA and saline was performed in this study. Furthermore, the present study has confirmed that the changes in MAP, HR and urinary protein concentration of this model were consistent with preeclampsia patients. In this study, the gestation period of rat is about 20–22 days. At 18 days after pregnant abdominal cavity space occupied by enlarged uterus of rats. To confirm the rat model of preeclampsia sympathetic never activity increased, we measured the content of NE in the 24 h urine. The content of NE was significantly higher in preeclampsia model group compared with Con groups, suggesting that sympathetic tone was increased in the rat with preeclampsia.

The balance between ROS generation and anti-oxidant mechanism determines the degree of oxidative stress and ROS (Espinosa-Diez et al., 2015; Matsubara et al., 2015; Venza et al., 2015). It has been reported that decrease in placental blood perfusion induces oxidation stress, vascular active factor imbalances, and endothelial cell injury, which plays an important role in the development and maintenance of preeclampsia (Jauniaux et al., 2006; Sedeek et al., 2008). Chappell et al. have also reported that supplementation with vitamins C and E, anti-oxidative vitamins, can be beneficial to preeclampsia in women at increased risk of the disease (Chappell et al., 1999). Indeed, it is well-known that ROS plays a key role in the mechanism of preeclampsia (D'Souza et al., 2016; Elliot, 2016). However, there is little study reported that whether the central oxidative stress has involved in the pathogenesis of preeclampsia, especially in oxidative stress in the RVLM. The enhanced oxidation stress contributes to the increase in sympathetic nerve activity and hypertension (Zimmerman et al., 2004; Oliveira-Sales et al., 2009). In the present study, we had observed the increased level of oxidative stress in the RVLM in preeclampsia rats. These results indicated that the increased generation of ROS in the RVLM might be involved in the pathogenesis of preeclampsia. It is suggested that the oxidative stress could increase the RVLM sympathetic outflow in the preeclampsia, in which there is an exaggerated super oxide anion generation. Moreover, it has demonstrated that microinjection of an antioxidant, vitamin C, into the RVLM resulted in a decrease in arterial pressure and sympathetic activity in hypertensive rats (Oliveira-Sales et al., 2008). In this work, microinjection of Tempol, a superoxide dismutase mimic, into the RVLM caused a significant decrease in BP, HR, and RSNA in preeclampsia rats but not in the control rats. In fact, this study was to determine whether oxidative stress was involved in cardiovascular abnormalities in preeclampsia, so acute microinjection of Tempol was to determine whether increased oxidative stress is involved in the maintenance of resting BP and sympathetic tone in preeclampsia state. We have realized that chronic infusion of Tempol is also very useful to detect the importance of oxidative stress in preeclampsia-associated high BP. This experiment would clarify if chronic decrease in oxidative stress improves the cardiovascular abnormalities induced by preeclampsia. Taken altogether, the results suggest that increased oxidative stress is involved in maintaining the cardiovascular activity in the preeclampsia.

There are many possible mechanisms involved in DOCA-induced increase in ROS in the RVLM. For example, DOCA can increase blood volume, which induces an increase in cardiac sympathetic afferent impulses, then causing changes in release of neurotransmitter in the central nervous system. It is reported that the central Ang II-associated oxidative stress was induced by activation of cardiac sympathetic afferent (Campese et al., 2005). To further explore the mechanism of ROS production in the RVLM in preeclampsia rats. It has been demonstrated that increased NADPH oxidase activity enhances ROS production, leading to hypertension (Oliveira-Sales et al., 2009). In this study, we had observed a significant increase in the NADPH oxidase subunits-NOX4 protein expression in the RVLM in preeclampsia rats. However, the superoxide dismutase subtype SOD1 expression in the RVLM in preeclampsia rats was unchanged compared to the Control groups. It is suggested that enhanced ROS production in the RVLM in preeclampsia rats is highly associated with the NADPH oxidase, especially NOX4 subunit. On the other hand, NADPH oxidase includes seven subtypes, NOX1, NOX2, NOX3, NOX4, NOX5, DUOX1, and DUOX2 (Bedard and Krause, 2007). Superoxide dismutase has main two subtypes, including SOD1 and SOD2 (Fukai and Ushio-Fukai, 2011). Our previous study has demonstrated that the ROS overproduction caused by upregulating protein expression of NOX4 and downregulating protein expression of SOD1 in the RVLM, which increased sympathetic tone in ovariectomized rats (Hao et al., 2016). In this work, there is a limitation that only the protein expressions of NOX4 subtype and SOD1 were detected. Further experiments are needed to determine whether other subtypes of NADPH oxidase and superoxide dismutase involved in the ROS production in the RVLM in preeclampsia.

In conclusion, our results had revealed that the level of ROS in the RVLM was enhanced in preeclampsia rats, and reduction in oxidative stress in the RVLM via microinjection of Tempol could ameliorate cardiovascular dysfunction in preeclampsia rats. Taken together, it is suggested that oxidative stress in the RVLM is involved in sympathetic overactivity and high BP, which contributes to the pathogenesis of preeclampsia.

## Author contributions

Study design: JY, MX. Performing experiments: JY, FangH, FanH, and XS. Data collection and analysis: JY, QM, and MX. Drafting manuscript: JY. Revising manuscript content: FangH and MX. Approving final version of manuscript: JY, FangH, FanH, XS, and MX.

### Conflict of interest statement

The authors declare that the research was conducted in the absence of any commercial or financial relationships that could be construed as a potential conflict of interest. The reviewer HW and handling Editor declared their shared affiliation.
